# Profil épidémiologique, clinique et évolutif des patients tuberculeux au Centre Hospitalier Régional (CHR) de Maradi, République du Niger

**DOI:** 10.11604/pamj.2019.33.120.17715

**Published:** 2019-06-17

**Authors:** Mahaman Laouali Harouna Amadou, Ousmane Abdoulaye, Oumarou Amadou, Ahamadou Biraïma, Sani Kadri, Abdoul Aziz Kabiru Amoussa, Ibrahim Maman Lawan, Laouali Tari, Maman Daou, Souleymane Brah, Eric Adehossi

**Affiliations:** 1 Service d’Infectiologie, Hôpital Régional de Maradi, Faculté des Sciences de la Santé de l’Université de Maradi, Maradi, Niger; 2 Service de Biologie Médicale, Hôpital Régional de Maradi, Faculté des Sciences de la Santé de l’Université de Maradi, Maradi, Niger; 3 Service de Médecine Interne, Hôpital Régional de Maradi, Faculté des Sciences de la Santé de l’Université de Maradi, Maradi, Niger; 4 Service d’Infectiologie, Hôpital Régional de Maradi, Maradi, Niger; 5 Centre Antituberculeux, Hôpital Régional de Maradi, Maradi, Niger; 6 Action Damien, Maradi, Niger; 7 Service de Médecine Interne, Hôpital National de Niamey, Faculté des Sciences de la Santé de l’UAM de Niamey, Niamey, Niger; 8 Service de Médecine Interne, Hôpital Général de Référence Niamey, Faculté des Sciences de la Santé de l’UAM de Niamey, Niamey, Niger

**Keywords:** Tuberculose, épidémiologie, clinique, évolution, Maradi, Niger, Tuberculosis, epidemiology, clinical, evolution

## Abstract

**Introduction:**

Il s'agissait de décrire le profil épidémiologique, clinique et évolutif des patients suivis pour tuberculose au Centre Hospitalier Régional de Maradi.

**Méthodes:**

Nous avions mené une étude rétrospective, descriptive et analytique à partir des dossiers des patients suivis pour tuberculose du 1^er^ janvier 2015 au 31 décembre 2017.

**Résultats:**

Au total 595 patients ont été suivis dont 406 hommes (68,24%) pour 189 femmes (31,76%) et une prévalence de 27,71%. L'âge moyen était de 42,3 ans avec des extrêmes de 13 mois à 85 ans. 70,5% des patients provenaient du milieu urbain. Les commerçants représentaient 36,9% des cas. La recherche bactériologique était positive dans 64,7% des cas. Les signes fonctionnels étaient représentés par: la toux (99,5%), la fièvre (79,5%), et la douleur thoracique. La tuberculose pulmonaire avait représenté 78,7%. Le succès thérapeutique a été 81,28%. La prévalence du VIH était de 13,6% et une létalité de 10,42% dont 40,4% des patients décédés avaient une co-infection TB/VIH.

**Conclusion:**

Avec 10,42% de décès, la tuberculose demeure un fléau dans les pays à faible revenu. L'infection à VIH/SIDA a négativement influencé ces décès au cours de cette étude. La recherche des facteurs de co-morbidités chez tout patient tuberculeux devrait être systématique afin d'améliorer leur prise en charge globale.

## Abstract (en)

**Introduction:**

This study aimed to describe the epidemiological, clinical and evolutionary profile of patients treated for tuberculosis at the Regional Hospital of Maradi.

**Methods:**

We conducted a retrospective, descriptive and analytical study of data from the medical records of patients treated for tuberculosis from 1^st^ January 2015 to 31^st^ December 2017.

**Results:**

A total of 595 patients were followed (406 men, 68.24%, and 189 women, 31.76%) with a prevalence of 27,71%. The average age of patients was 42.3 ranging from 13 months to 85 years; 70.5% of these patients were from urban areas. Merchants represented 36.9% of the cases. Bacterial test was positive in 64.7% of cases. Functional signs included: coughing (99.5%), fever (79.5%), and chest pain. Pulmonary tuberculosis represented 78.7% of cases. Therapy was effective in 81.28% of cases. HIV prevalence was 13.6%, lethality 10.42% (40.4% of patients died from TB/HIV co-infection).

**Conclusion:**

Tuberculosis is a scourge in low-income countries, with 10.42% of deaths. HIV/AIDS infection has negatively contributed to these deaths during the study period. The search for comorbidities in any patient with tuberculosis should be systematic in order to improve their global management.

## Introduction

La tuberculose est une maladie infectieuse contagieuse provoquée par une mycobactérie du complexe *tuberculosis* principalement le *Mycobacterium tuberculosis* ou bacille de Koch [1]. Elle constitue aujourd'hui un problème majeur de santé publique au niveau mondial. D'après les dernières estimations de l'OMS, il y a environ 10,4 millions de cas de tuberculose à l'échelle mondiale dont 90% d'adultes, 65% de sexe masculin, et 10% vivant avec le VIH [2]. En Afrique, le taux de décès estimé à 81 pour 100.000 habitants est le plus élevé au monde [3]. Le but de cette étude était de déterminer le profil épidémiologique, clinique et évolutif des patients suivis pour tuberculose pharmaco-sensible au centre anti tuberculeux du CHR de Maradi.

## Méthodes

Nous avions mené une étude rétrospective, descriptive et analytique basée sur l'examen des dossiers des patients suivis pour tuberculose au centre antituberculeux (CAT) du CHR de Maradi, au cours de la période allant du 1^er^ janvier 2015 au 31 décembre 2017 soit trois ans. Cette étude avait concerné une cohorte de patients suivis au niveau du CAT sans distinction d'âge ni de sexe. Les patients inclus étaient ceux qui avaient un diagnostic de tuberculose (recherche bactériologique concluante et cliniquement diagnostiqué). Etaient exclus, ceux dont le dossier est inexploitable ou inexistant. Le diagnostic de la tuberculose avait été retenu sur les arguments cliniques, paracliniques et épidémiologie suggestives. Les données recueillies étaient analysées grâce aux logiciels Word 2007 et Epi Info.

## Résultats

Durant la période de l'étude, 16.493 patients étaient consultés au CHR de Maradi dont 595 pour une tuberculose soit 3,6%. Le Tableau 1, résume les caractéristiques épidémiologiques de notre population d'étude. Sur le plan clinique, les signes fonctionnels étaient représentés par la toux et la fièvre. La sérologie VIH était positive chez 81 patients (TB/VIH+), soit un taux de séroprévalence VIH de 13,61% (Tableau 2). Sur le plan évolutif, le succès thérapeutique était de 81,28%, et 10,42% de décès (Tableau 3). On avait noté par ailleurs que 40% des décès étaient liés à la co-infection TB/VIH.

**Tableau 1:** Caractéristiques épidémiologiques

Variables	Effectif	%
Age (ans)		
˂ 20	58	9,74
21 - 40	340	57,14
41- 60	162	27,22
˃ 60	35	5,9
Age moyen (extrêmes)	42,3± 10,3 ans (13 mois à 85 ans)	
Sexe		
Hommes	406	68,24
Femmes	189	31,76
Sex ratio	2,14	
Lieu de résidence		
Centre-ville	419	70,5
Villages environs	176	29,5

**Tableau 2:** Caractéristiques cliniques et paracliniques

Variables	Fréquence	%
Toux	592	99,5
Dyspnée	255	42,9
Douleur thoracique	315	53,02
Hémoptysie	20	3,3
Fièvre	473	79,5
Sérologie VIH positif	81	13,61
Recherche de BAAR positif	385	64,7

BAAR= bacilles acido-alcoolo-résistants

**Tableau 3:** Caractéristiques évolutifs

Variables	Fréquence	%
Succès thérapeutique	484	81,28
Echec thérapeutique	5	0 ,9
Perdu de vue	44	7,4
Décédés	62	10,42

## Discussion

Ce travail portant sur la tuberculose au Centre Hospitalier Régional de Maradi (sud du Niger) a montré 595 cas dont 62 décès où peu de publications existent sur cette maladie, justifiant ainsi ce travail. Notre étude était rétrospective, portant sur les dossiers des patients suivis durant la période allant du 1^er^ janvier 2015 au 31 décembre 2017 soit trois ans. Ainsi, certaines informations telles que, la notion de contage ou les effets secondaires des antituberculeux, les transferts des patients étaient manquantes dans la plupart des dossiers et n'ont pu être prises en compte dans notre travail. L'incidence de la tuberculose est variable dans le monde allant d'une incidence faible entre 0 et 9,9 pour 100 000 habitants par an dans les pays occidentaux (Amérique du Nord et Europe), à des incidences extrêmement élevées, supérieures à 500 pour 100 000 habitants par an en Afrique du sud [1]. Le Niger est un pays à haute endémicité tuberculeuse avec un taux d'incidence estimé à 98 nouveaux cas pour 100.000 habitants [4]. Au Togo pays proche du Niger, ce taux était de 73 cas pour 100.000 habitants en 2012 [5]. Dans notre étude, les hommes (68,24%) sont plus affectés que les femmes (31,76%). Cette prédominance masculine a été rapportée par d'autres auteurs [6-8]. En revanche David Lupande *et al*. et Esthel Lee Presley Bemba *et al*. en RD Congo ont rapporté une prédominance féminine de la tuberculose dans leurs études [9, 10]. L'âge moyen de nos patients est de 42,3 ± 10,1 ans avec des extrêmes de 13 mois à 85 ans ce qui confirme le caractère contagieux de cette maladie, donc touchant les sujets à tout âge en fonction de leur exposition. Les malades venant du centre-ville sont les plus nombreux (70,5%), ceci est dû au simple fait que le lieu de cette étude est implanté en plein coeur de la commune urbaine de Maradi, mais aussi par ce que c'est un centre de référence de prise en charge de tuberculose pour toute la région. Sur le plan clinique nous avons retrouvés la toux (99,5%), la douleur thoracique (53,02%), la dyspnée (42,9%), l'hémoptysie (3,3%) et la fièvre (79,5%) qui sont des signes confirmant les données de la littérature [1, 8, 11-13]. Dans notre série 78,7% de nos patients avaient présenté la tuberculose pulmonaire. Ce constat a été fait par beaucoup d'autres auteurs notamment Yombi et Olinga en Belgique qui ont rapporté 70 à 75% des cas [1], Breton G *et al*. en République Centrafricaine qui ont rapporté respectivement pour les patients VIH+ et VIH- 83% et 79 % [14], Shimazaki *et al*. à Manille au Philippines qui ont rapporté 82,4% [12] et Ouedraogo *et al*. au Niger qui ont rapporté 44,9% [15].

Sur un total de 595 patients nous avons enregistrés 385 cas de tuberculose confirmés par le laboratoire d'analyse de biologie médicale du CHR soit 64,7%. Christian Kakisingi Ngama *et al*. en RD Congo ont rapporté un taux de 31,9% [6], inférieur à celui de notre étude. Par contre Diallo *et al*. au Mali, ont rapporté un taux de 73,9% [16], supérieur à celui de notre étude. Touré *et al*. au Sénégal ont rapporté dans leur série respectivement 91,6 et 83,4% de tuberculose bactériologiquement confirmés pour les sujets âgés et jeunes. Parmi nos patients, 81 avaient une sérologie au virus de l'immunodéficience acquise positive soit 13,61%, Dagnra *et al*. dans leur série au Togo avaient rapporté sur 569 patients, 135 (23,7%) des patients tuberculeux étaient infectés par le VIH. Quant aux Breton *et al*. en Centrafrique, ils avaient rapporté un taux très élevé par rapport à celui de notre étude 82% [14], cela pourrait s'expliquer par le fait que les taux les plus élevés de l'infection à VIH chez les patients tuberculeux en Afrique, sont observés en Afrique Australe et Centrale [17], mais également par le fait que l'infection à VIH favorise la réactivation d'une infection latente à *M. tuberculosis* vers une TB maladie et *M. tuberculosis* favorise la réplication du VIH et accélère ainsi l'évolution naturelle de l'infection vers une immunodepression [18, 19]. L'un des indicateurs de l'évaluation de performance des Programmes nationaux de lutte contre la TB est le taux de guérison. Pour l'OMS, le taux de guérison recommandé pour les pays à ressources limitées est de 85 % des malades tuberculeux nouveaux cas dépistés. Dans cette étude, le taux de guérison était de 81,2 8%, donc proche des recommandations de l'OMS et semblable à celui recommandé au plan national qui d'environ 80% [4]. Par contre il était supérieur aux conclusions de Dye *et al*, selon lesquelles le taux de guérison global de la TB varie de 71,1 à 74,4 % en Afrique, en fonction de la prévalence de l'infection à VIH [20]. Nous avions enregistrés 44 perdus de vue soit un taux de 7,4% superposable à celui rapporté par le Programme National de Lutte Contre la Tuberculose (PNLT) dans leur plan stratégique 2019-2021 [4], mais inférieur aux résultats de Segbedji *et al*. au Togo [5]. Le taux de mortalité hospitalière chez nos patients est de 10,42%, supérieur à celui rapporté par une étude réalisée au plan national par le PNLT qui est de 7% [4], mais inférieur à celui rapporté par Shimazaki *et al*. à Manille au Philippines qui ont rapporté 37,5% [12].

## Conclusion

Dans notre étude, sur 595 patients enregistrés pour tuberculose, 484 sont guéris soit 81,28%. La tuberculose est encore d’actualité, touchant toutes les tranches d'âges surtout dans les pays en développement. La co-infection tuberculose-VIH est un facteur aggravant la mortalité. L'éradication de la tuberculose passera par une prise en charge adéquate basée sur un dépistage précoce des cas et une diminution du taux des perdus de vue à travers une éducation thérapeutique des patients favorisant leur parfaite adhérence au traitement.

### Etat des connaissances actuelles sur le sujet

- De nos jours la tuberculose demeure un fléau dans les pays à faible revenu comme le Niger marqué par l'impact du VIH/SIDA;
- Les mauvaises prescriptions, les traitements empiriques inappropriés, l'automédication, l'inobservance du traitement entrainent la sélection et le maintien des souches mycobactériennes multi-résistantes.

### Contribution de notre étude à la connaissance

- Prévalence de la tuberculose: la présente étude décrit la prévalence de la tuberculose dans la région de Maradi au Niger;
- Influence du VIH: l'infection à VIH/SIDA a négativement influencé ces décès au cours de cette étude;
- Importance de la recherche des facteurs de co-morbidités: la recherche des facteurs de co-morbidités chez tout patient tuberculeux devrait être systématique afin d'améliorer leur prise en charge globale.

## Conflits d’intérêts

Les auteurs ne déclarent aucun conflit d'intérêts.
